# Self-Reported Prevalence and Risk Factors for Shaking and Smothering Among Mothers of 4-Month-Old Infants in Japan

**DOI:** 10.2188/jea.JE20140216

**Published:** 2016-01-05

**Authors:** Takeo Fujiwara, Yui Yamaoka, Naho Morisaki

**Affiliations:** 1 Department of Social Medicine, National Research Institute for Child Health and Development, Tokyo, Japan; 1 国立成育医療研究センター研究所社会医学研究部; 2 Department of Developmental Social Medicine, Mie University Graduate School of Medicine, Tsu, Japan; 2 三重大学大学院医学系研究科成育社会医学分野; 3 Department of Health Services Research, Faculty of Medicine, University of Tsukuba, Tsukuba, Ibaraki, Japan; 3 筑波大学医学医療系ヘルスサービスリサーチ分野

**Keywords:** abusive head trauma, child abuse, crying, shaken baby syndrome, shaking, smothering, Japan

## Abstract

**Background:**

To estimate the prevalence of shaking and smothering and to determine risk factors in a population-based sample of mothers with 4-month-old infants in Japan.

**Methods:**

We administered a questionnaire to women who participated in a 4-month health checkup program in Aichi Prefecture, Japan (*n* = 6487; valid response rate, 66.8%), and assessed frequency of shaking and smothering during the past 1 month, as well as maternal, infant, and familial characteristics. Associations of shaking, smothering, and either shaking or smothering with possible risk factors were analyzed using multiple logistic regression.

**Results:**

Self-reported prevalence of shaking, smothering, and either shaking or smothering at least once during the past month was 3.9% (95% confidence interval [CI], 3.5%–4.4%), 2.7% (95% CI, 2.3%–3.1%), and 5.4% (95% CI, 4.9%–6.0%) respectively. Several different risk factors were found for shaking and smothering. Risk factors for either shaking or smothering were age 34 years or younger (especially 24 years or younger), age 40 years or older, full-time working, later attendance at 4-months health checkup, primiparity, living in a detached house, living on the 2nd floor or higher (especially on the 10th floor or higher), economic adversity, perceived excessive crying, and postpartum depression. Protective factors against infant abuse were living in a four-room house and having a larger number of people to consult with.

**Conclusions:**

Self-reported prevalences of shaking and smothering among mothers in Japan were similar to prevalences reported in western countries. These finding may be useful for identifying mothers at increased risk of shaking and smothering their infants.

## Abstract (ja)

**背景：:**

日本の人口ベースのサンプルを用いて、4か月の乳児をもつ母親の揺さぶりと口塞ぎの存在割合を推定し、リスク因子を明らかにすること。

**方法：:**

愛知県45市の4か月健診を受診した母親に質問紙調査を実施した(N=6,487, 有効回答率: 66.8%)。その質問紙で、過去1か月間の揺さぶりおよび口塞ぎの頻度、母親・子の特性、世帯の状況について調査した。そして、これらリスク因子と揺さぶり、口塞ぎ、揺さぶりまたは口塞ぎとの関連をロジスティック回帰分析によって検討した。

**結果：:**

自己申告による過去1か月の1回以上の揺さぶり、口塞ぎ、そのどちらかの存在割合は、それぞれ3.9%（95%信頼区間：3.5%-4.4％）、2.7%（95%信頼区間：2.3%-3.1%), 5.4% (95% 信頼区間：, 4.9%-6.0%)であった。揺さぶりまたは口塞ぎのリスク因子は、母親の年齢が34歳以下（特に24歳以下）または40歳以上、母親が常勤で勤務していること、4か月健診の受診月齢が遅いこと、初産、一戸建て居住、２階以上の集合住宅（特に１０階以上の集合住宅）の居住、主観的経済状況の悪さ、泣きの量を多いと感じること、産後うつ、であった。保護因子としては部屋の数が４つあること、そして相談できる人の数が多いこと、であった。

**結論：:**

日本における自己申告の揺さぶりおよび口塞ぎの存在割合は欧米諸国と同等であった。この研究結果が、揺さぶりや口塞ぎといった乳児虐待のリスクを持つ母親を特定することに役立つかもしれない。

## INTRODUCTION

Shaken baby syndrome (SBS) or abusive head trauma (AHT) is the leading cause of death due to child abuse.^1^ To prevent SBS/AHT, the risk factors for shaking need to be determined. Well-known risk factors for shaking include frustration due to inconsolable crying,^2^ young infant age,^3^^,^^4^ young maternal age, multiple births, having a male infant,^5^ and economic adversity.^6^ However, other potential risk factors, such as living environment, have not been well described.

When discussing SBS/AHT, definition is crucial. Adamsbaum et al reported that, in more than half of cases admitted for SBS, the infant had been shaken by the perpetrator on at least one prior occasion.^7^ This suggests that the ascertainment of SBS/AHT cases could occur before caregivers shake their infant repeatedly and severely enough to manifest clinical symptoms that lead to hospitalization. As such, it is important to understand shaking behavior that occurs prior to clinical assessment. Therefore, it is necessary to identify the characteristics of mothers who are at a high risk of shaking their infant using a community sample. In a Dutch study,^8^ 1.3% and 3.4% of parents of 3- and 6-month-old infants, respectively, reported that they had shaken their infants at least once. In this study, we aim to identify prevalence of shaking and its risk factors in Japan, where housing in relatively small and high-rise apartment complexes are common.^9^ Such living arrangements might contribute to increased maternal frustration about crying and may be a factor in infant shaking.

Like shaking, smothering is also a form of life-threatening child abuse^10^^,^^11^; however, its prevalence and risk factors have not been widely reported. In the Netherlands, the cumulative rate of self-reported smothering was reported by 1.6% of caregivers of 6-month-old infants.^8^ However, the prevalence of smothering is of interest in Japan because Japanese caregivers tend to be anxious that an infant’s crying may disturb cohabitants (eg, grandparents) or neighbors in close proximity.^12^ As with shaking, Japan’s dense, small apartments may also contribute to the risk of smothering.

The purpose of this study was to estimate self-reported prevalences of shaking and smothering and to determine risk factors in a population-based sample of mothers of 4-month-old infants in Japan.

## METHODS

### Sample

All 54 municipalities in Aichi Prefecture were invited to participate, and 45 municipalities, including Nagoya City—the capital city of Aichi Prefecture—agreed to join this study. Aichi Prefecture is located between Tokyo and Osaka in Japan and had a population of approximately 7.4 million with 67 913 births in 2012. The combined population of the participating municipalities covers 80% of Aichi Prefecture’s total population. The target subjects were all mothers (*n* = 9707) who were enrolled in a 3- or 4-month health checkup program between October and November 2012 in participating municipalities. An anonymous questionnaire was mailed directly to the target women before the start of the health checkup program, and responses were collected during each health checkup in 34 municipalities. In the remaining 11 municipalities, questionnaires were distributed during the 3- or 4-month health checkup, and the participants’ responses were returned by post to each health center. Overall, the participation rate for the 3- or 4-month health checkup in Aichi Prefecture was 97.9%. In total, 6590 women responded (response rate, 68%; range among municipalities, 24.2%–81.0%). Our study was approved by the ethics committee of the National Center for Child Health and Development, which determined that it was unnecessary to obtain consent from participants given that responding to the anonymous questionnaire already implied consent to participate in the study.

### Shaking and smothering measures

The questionnaire assessed whether participants had shaken or smothered their infant in the past 1 month. The following question was asked (in Japanese) about frequency of shaking behavior: “When your child is crying and making a scene, how many times have you violently shaken your child in the past 1 month?” with possible responses of “0 times,” “1 or 2 times,” “3–5 times,” “6–10 times,” and “11 or more times.” Because the term ‘shaking’ may be misunderstood as ‘rocking’ in Japanese, we instead used the Japanese term for ‘violently shaking’ in the questionnaire. The following question was specifically asked about smothering: “How many times have you ever covered the mouth of your baby when crying, using your hands, a cushion, etc in the past 1 month?” The same response items as those for shaking were used to assess smothering frequency.

### Risk factors

The questionnaire covered the following five areas: parental demographics (maternal and paternal age, marital status, and maternal employment status), obstetric history (experience of miscarriage, induced abortion, and fertility treatment), infant characteristics (age in weeks, sex, birth weight, gestational age, multiple births, delivery method [vaginal or cesarean delivery], and birth order), household characteristics (living with grandparents and housing type [ie, a detached house or apartment complex]), number of rooms, and subjective socioeconomic status (4-point Likert response items [stable, able to manage, difficult to manage, or unstable]), and postpartum situation around 4 months (feeding status, number of persons to consult with, perceived amount of infant crying, and postpartum depression [assessed using the Edinburgh Postnatal Depression Scale {EPDS}]^13^). Perceived amount of infant crying was assessed based on the response to the question, “Does your baby cry a lot?” using a 4-point Likert scale, with 1 indicating “not at all” and 4 indicating “yes, a lot.” Following the results of a previous community study in Japan, we defined postpartum depression as having an EPDS score of 9 or higher.^14^ Regarding subjective economic situation, due to the low percentage of respondents choosing “unstable” (2.5%), “difficult to manage” and “unstable” were collapsed for further analysis. Similarly, the responses on perceived amount of crying of “a lot” and “to some extent” were also collapsed, due to the low percentage of respondents choosing “a lot” (5.1%).

### Analysis

We excluded questionnaires that contained a non-valid response to questions about shaking and smothering (*n* = 103), resulting in a sample size of 6487 women. Shaking or smothering responses were dichotomized, with a frequency of zero times defined as a “no” response and a frequency of one or more times defined as a “yes” response. The associations between possible risk factors and shaking, smothering, and either shaking or smothering were analyzed using multiple logistic regression. In addition to an initial bivariate model, we calculated a multivariate model (model 1) that adjusted for parental demographics (maternal age, marital status, and maternal employment status), obstetric history (miscarriage, induced abortion, and fertility treatment), infant characteristics (age in weeks, sex, birth weight, gestational age, multiple birth or not, delivery method, and birth order), and household characteristics (living with grandparents or not, house type [ie, detached house or apartment, and, if apartment, level of living floor], number of rooms, and subjective socioeconomic status), and another multivariate model (model 2) that adjusted for the postpartum situation at 4 months (feeding status, number of persons to consult with, perceived frequent infant crying, and postpartum depression), in addition to the covariates included in model 1. Paternal age was not used due to multicollinearity with maternal age. For analysis of the association with either shaking or smothering, we further stratified by region (Nagoya City or other cities). All analyses were conducted using Stata/MP v12.0 software (StataCorp, College Station, TX, USA).

## RESULTS

Participant characteristics are presented in Table 1. Most women were 25–39 years old (87.5%), married (98.6%), and not working (78.0%). Most infants were aged 13–20 weeks, but some visited the clinic for their health checkup earlier (5.3%) or later (2.2%) and so were slightly younger or older. Around 13% of infants were living with grandparents, and 58% were living in an apartment complex. Regarding economic status, 11% reported that their finances were difficult to manage or unstable. A total of 23% of mothers perceived their infant’s crying as “a lot” or “to some extent”. Participants considered at risk of having postpartum depression (ie, an EPDS score ≥9) represented 9.5% of the sample. In Nagoya City, 3.8% of women lived on the 10th floor or higher, compared to 1.0% in other cities (*P* for chi-square <0.01). Further, the percentage of women living with grandparents was lower in Nagoya City than other cities (9.4% vs 15.2%, *P* for chi-square <0.01).

**Table 1.** Characteristics of sample

		Total (*n* = 6487)		Nagoya City (*n* = 2575)		Other cities (*n* = 3912)	
		
		*n*	%	*n*	%	*n*	%
**Parental demographics**							
Maternal age, years	≤19	33	0.5	9	0.4	24	0.6
	20–24	475	7.3	146	5.7	329	8.4
	25–29	1792	27.6	691	26.8	1101	28.1
	30–34	2413	37.2	999	38.8	1414	36.2
	35–39	1475	22.7	592	23.0	883	22.6
	≥40	285	4.4	133	5.2	152	3.9
	Missing	14	0.2	5	0.2	9	0.2
Paternal age, years	≤19	13	0.2	4	0.2	9	0.2
	20–24	288	4.4	82	3.2	206	5.3
	25–29	1318	20.3	498	19.3	820	21.0
	30–34	2212	34.1	879	34.1	1333	34.1
	35–39	1774	27.4	700	27.2	1074	27.5
	≥40	815	12.6	380	14.8	435	11.1
	Missing	67	1.0	32	1.2	35	0.9
Marital status	Married/living with partner	6396	98.6	2532	98.3	3864	98.8
	Single/divorced/widowed	75	1.2	35	1.4	40	1.0
	Missing	16	0.3	8	0.3	8	0.2
Maternal employment status	Not working	5061	78.0	2036	79.1	3025	77.3
	Full-time	1075	16.6	416	16.2	659	16.9
	Part-time	316	4.9	109	4.2	207	5.3
	Missing	35	0.5	14	0.5	21	0.5
**Obstetrics history**							
Miscarriage	Yes	1155	17.8	449	17.4	706	18.1
Induced abortion	Yes	447	6.9	182	7.1	265	6.8
Fertility treatment	Yes	747	11.5	277	10.8	470	12.0
**Infant characteristics**							
Age, weeks	≤12	341	5.3	135	5.2	206	5.3
	13–20	5594	86.2	2237	86.9	3357	85.8
	≥21	142	2.2	21	0.8	121	3.1
	Missing	410	6.3	182	7.1	228	5.8
Sex	Boy	3268	50.4	1279	49.7	1989	50.8
	Girl	3159	48.7	1269	49.3	1890	48.3
	Missing	60	0.9	27	1.1	33	0.8
Birth weight, grams	<2500	540	8.3	221	8.6	319	8.2
	≥2500	5920	91.3	2344	91.0	3576	91.4
	Missing	27	0.4	10	0.4	17	0.4
Gestational age, weeks	<37	375	5.8	137	5.3	238	6.1
	≥37	5980	92.2	2396	93.1	3584	91.6
	Missing	132	2.0	42	1.6	90	2.3
Multiple births	Single	6380	98.4	2529	98.2	3851	98.4
	Twin	107	1.7	46	1.8	61	1.6
Delivery method	Vaginal	5111	78.8	2001	77.7	3110	79.5
	Cesarean	1325	20.4	549	21.3	776	19.8
	Missing	51	0.8	25	1.0	26	0.7
Birth order	First child	3203	49.4	1303	50.6	1900	48.6
	Subsequent child	3284	50.6	1272	49.4	2012	51.4
**Household characteristics**							
Living with grandparents	Yes	838	12.9	243	9.4	595	15.2
	No	5649	87.1	2332	90.6	3317	84.8
House type	Detached house	2534	39.1	646	39.1	1888	48.3
	Apartment, 1st (ground) floor	935	14.4	340	14.4	595	15.2
	Apartment, 2nd–9th floor	2723	42.0	1418	55.1	1305	33.4
	Apartment, ≥10th floor	136	2.1	99	2.1	37	1.0
	Missing	159	2.5	72	2.5	87	2.2
Number of rooms	1–2 rooms	858	13.2	371	14.4	487	12.5
	3 rooms	2173	33.5	911	35.4	1262	32.3
	4 rooms	1415	21.8	704	27.3	711	18.2
	≥5 rooms	1817	28.0	526	20.4	1291	33.0
	Missing	224	3.5	63	2.5	161	4.1
Subjective economic status	Stable	2876	44.3	1169	45.4	1707	43.6
	Able to manage	2655	40.9	998	38.8	1657	42.4
	Difficult to manage or unstable	738	11.4	304	11.8	434	11.1
	Missing	218	3.4	104	4.0	114	2.9
**Postpartum situation**							
Feeding status at 4 months	Breastfeeding only	3882	59.8	1532	59.5	2350	60.1
	Mixed	1489	23.0	568	22.1	921	23.5
	Bottle-feeding only	709	10.9	262	10.2	447	11.4
	Missing	407	6.3	213	8.3	194	5.0
Number of persons to consult with	0–5 persons	2494	38.5	1009	39.2	1485	38.0
	6–10 persons	2529	39.0	982	38.1	1547	39.5
	≥11 persons	1066	16.4	427	16.6	639	16.3
	Missing	398	6.1	157	6.1	241	6.2
Perception of frequency of infant crying	Not at all	2048	31.6	823	32.0	1225	31.3
	Not so much	2919	45.0	1146	44.5	1773	45.3
	A lot or to some extent	1492	23.0	598	23.2	894	22.9
	Missing	28	0.4	8	0.3	20	0.5
Postpartum depression EPDS score	≥9	618	9.5	229	8.9	389	9.9
	≤8	5849	90.2	2340	90.9	3509	89.7
	Missing	20	0.3	6	0.2	14	0.4

Table 2 shows the association of the prevalence of shaking and smothering frequencies. The overall prevalence of shaking at least once during the past 1 month was 3.9% (95% confidence interval [CI], 3.5%–4.4%). The overall prevalence of smothering at least once during the past 1 month was 2.3% (95% CI, 1.5%–3.1%), and the prevalence of infant abuse (ie, either shaking or smothering) was 5.4% (95% CI, 4.9–6.0%). We also found a high comorbidity rate: 80 cases out of 255 shaking mothers also smothered their infant (31.4%), and 80 cases out of 178 smothering mothers also shook their infant (44.9%).

**Table 2.** Association of prevalence of shaking and smothering (*n* = 6487)

		Smothering		

		0 times	≥1 times	Total
Shaking	0 times	6134 (94.56)	98 (1.51)	6232 (96.07)
	≥1 times	175 (2.70)	80 (1.23)	255 (3.93)

	Total	6309 (97.26)	178 (2.74)	6487 (100)

Odds ratios (ORs) of possible risk factors for shaking at least once during the past 1 month are shown in Table 3. Maternal age <29 years (compared to those aged 35–39 years), maternal full-time work, no history of miscarriage, later attendance at the 4-month health checkup (compared to those who attended when their children were aged 13–20 weeks), and primiparity were associated with shaking, while child’s sex, low birth weight, being a preterm birth, being a multiple birth, and delivery method were not associated with shaking. Regarding living environment, mothers living on the 10th floor of an apartment complex or higher were 3.47 (95% CI, 1.48–8.15) times more likely than mothers living on the ground floor to have shaken their infants. Further, mothers living in a home with four rooms were less likely to shake their infants than mothers living in a three-room home, even after adjustment for economic status and postpartum situation (OR 0.58; 95% CI, 0.38–0.88). Economic adversity, mixed feeding (in comparison with breastfeeding only), a perceived larger amount of infant crying, and postpartum depression were also independently and significantly associated with shaking.

**Table 3.** Odds ratios of parental demographics, obstetrics history, infant characteristics, household characteristics, and postpartum situation for shaking at 4 months of age

		Prevalence of shaking (%)	Bivariate		Model 1^a^		Model 2^b^	
		
			OR	95% CI	aOR	95% CI	aOR	95% CI
**Parental demographics**								
Maternal age, years	≤19	12.1	**5.67**	**1.89–17.0**	**3.49**	**1.08–11.3**	**3.54**	**1.08–11.6**
	20–24	9.3	**4.20**	**2.66–6.63**	**3.30**	**2.01–5.41**	**3.53**	**2.13–5.84**
	25–29	4.3	**1.85**	**1.23–2.77**	1.49	0.97–2.29	**1.65**	**1.07–2.55**
	30–34	3.4	1.45	0.97–2.16	1.32	0.88–1.99	1.49*	0.99–2.26
	35–39	2.4	Ref		Ref		Ref	
	≥40	3.9	1.65	0.83–3.29	1.68	0.83–3.39	1.63	0.80–3.33
Marital status	Married/living with partner	3.9	Ref		Ref		Ref	
	Single/divorced/widowed	8.0	2.16	0.93–5.03	1.54	0.62–3.85	1.44	0.58–3.58
Maternal employment status	Not working	3.8	Ref		Ref		Ref	
	Full-time	4.5	1.18	0.85–1.63	1.36	0.97–1.90	**1.45**	**1.03–2.05**
	Part-time	3.2	0.82	0.43–1.57	0.85	0.44–1.64	0.90	0.46–1.75
**Obstetrics history**								
Miscarriage	No	4.3	Ref		Ref		Ref	
	Yes	2.3	**0.51**	**0.34–0.77**	**0.61**	**0.40–0.94**	**0.61**	**0.40–0.93**
Induced abortion	No	3.9	Ref		Ref		Ref	
	Yes	4.7	1.22	0.77–1.93	1.11	0.69–1.78	1.12	0.69–1.81
Fertility treatment	No	4.0	Ref		Ref		Ref	
	Yes	3.5	0.87	0.57–1.31	1.05	0.68–1.63	1.00	0.64–1.56
**Infant characteristics**								
Age, weeks	≤12	5.6	1.53	0.94–2.48	1.58	0.96–2.58	1.62	0.98–2.70
	13–20	3.7	Ref		Ref		Ref	
	≥21	9.2	**2.61**	**1.45–4.69**	**2.70**	**1.47–4.96**	**2.75**	**1.47–5.15**
Sex	Boy	4.1	Ref		Ref		Ref	
	Girl	3.7	0.88	0.69–1.14	0.90	0.70–1.17	0.98	0.75–1.27
Birth weight, grams	<2500	5.4	1.44	0.97–2.14	1.37	0.86–2.18	1.24	0.77–1.99
	≥2500	3.8	Ref		Ref		Ref	
Gestational age, weeks	<37	5.3	1.42	0.89–2.27	1.23	0.71–2.13	1.05	0.60–1.83
	≥37	3.8	Ref		Ref		Ref	
Multiple births	Single	3.9	Ref		Ref		Ref	
	Twin	4.7	1.20	0.49–2.98	1.05	0.37–2.94	0.92	0.32–2.62
Delivery method	Vaginal	4.1	Ref		Ref		Ref	
	Cesarean	3.3	0.79	0.57–1.10	0.81	0.57–1.16	0.80	0.56–1.14
Birth order	First child	5.0	**1.73**	**1.34–2.45**	**1.51**	**1.14–2.00**	1.29	0.97–1.72
	Subsequent child	2.9	Ref		Ref		Ref	
**Household characteristics**								
Living with grandparents	Yes	3.5	0.86	0.58–1.28	0.72	0.46–1.14	0.69	0.44–1.09
	No	4.0	Ref		Ref		Ref	
House type	Detached house	3.5	0.95	0.64–1.43	**1.80**	**1.06–3.04**	**1.77**	**1.03–3.04**
	Apartment, 1st (ground) floor	3.6	Ref		Ref		Ref	
	Apartment, 2nd–9th floor	4.5	1.24	0.84–1.83	**1.50**	**1.01–2.24**	1.48*	0.99–2.22
	Apartment, ≥10th floor	5.9	1.66	0.75–3.66	**3.17**	**1.37–7.29**	**3.47**	**1.48–8.15**
Number of rooms	1–2 rooms	4.9	1.03	0.72–1.49	0.92	0.63–1.34	0.91	0.62–1.34
	3 rooms	4.7	Ref		Ref		Ref	
	4 rooms	2.6	**0.54**	**0.37–0.79**	**0.56**	**0.37–0.85**	**0.58**	**0.38–0.88**
	≥5 rooms	3.1	**0.65**	**0.47–0.91**	0.73	0.45–1.17	0.77	0.48–1.26
Subjective economic status	Stable	3.0	Ref		Ref		Ref	
	Able to manage	4.3	**1.44**	**1.08–1.92**	**1.40**	**1.04–1.88**	1.17	0.86–1.58
	Difficult to manage or unstable	6.2	**2.16**	**1.49–3.11**	**2.08**	**1.41–3.06**	**1.60**	**1.07–2.40**
**Postpartum situation**								
Feeding status at 4 months	Breastfeeding only	3.1	Ref				Ref	
	Mixed	4.8	**1.59**	**1.18–2.15**			**1.38**	**1.01–1.88**
	Bottle-feeding only	5.6	**1.87**	**1.30–2.71**			1.47*	0.99–2.18
Number of persons to consult with	0–5 persons	5.1	Ref				Ref	
	6–10 persons	3.2	**0.62**	**0.47–0.83**			0.75*	0.55–1.00
	≥11 persons	2.8	**0.54**	**0.36–0.81**			0.74	0.49–1.13
Perception of frequency of infant crying	Not at all	1.5	Ref				Ref	
	Not so much	4.0	**2.69**	**1.80–4.02**			**2.73**	**1.81–4.11**
	A lot or to some extent	7.2	**5.08**	**3.39–7.61**			**4.64**	**3.05–7.07**
Postpartum depression EPDS score	≥9	3.4	**2.76**	**2.02–3.76**			**1.95**	**1.38–2.75**
	≤8	8.9	Ref				Ref	

CI, confidence interval; OR, odds ratio; Ref, reference.

Bold signifies *P* < 0.05.

*P < 0.06.

^a^Model 1 adjusted for parental demographics (maternal age, marital status, and maternal employment status), obstetric history (miscarriage, induced abortion, and fertility treatment), infant characteristics (age in weeks, sex, birth weight, gestational age, multiple birth or not, delivery method, and birth order), and household characteristics (living with grandparents or not, house type, that is, detached house or apartment, and if apartment, level of living floor, number of rooms, and subjective socioeconomic status).

^b^Model 2 adjusted for covariates in Model 1 plus postpartum situation at 4 months (feeding status, number of persons to consult with, perception of frequent infant crying, and postpartum depression).

ORs of possible risk factors for smothering at least once during the past 1 month are shown in Table 4. As with shaking, younger mothers (<19 or 20–24 years old) were more likely to smother their infants than those aged 35–39 years (OR 8.54; 95% CI, 2.82–25.9 and OR 2.36; 95% CI, 1.26–4.42, respectively). Infant characteristics conducive to smothering were the same to those conducive to shaking (later attendance at 4-month health checkup and primiparity). In terms of the living environment, mothers living on the 10th floor or higher and on the 2nd to 9th floor of an apartment complex were 5.90 (95% CI, 2.38–14.6) and 2.00 (95% CI, 1.20–3.35) times more likely to smother their infants than mothers living on the ground floor. In contrast to shaking, the number of rooms, subjective socioeconomic status, and feeding type were not associated with smothering. As with shaking, perceived larger amount of infant crying and postpartum depression were both independently and significantly associated with smothering. Further, we found that having 6–10 persons to consult with compared to 0–5 persons was a significant protective factor against smothering (OR 0.65; 95% CI, 0.46–0.93).

**Table 4.** Odds ratios of parental demographics, obstetrics history, infant characteristics, household characteristics, and postpartum situation for smothering at 4 months of age

		Prevalence of smothering (%)	Bivariate		Model 1^a^		Model 2^b^	
		
			OR	95% CI	aOR	95% CI	aOR	95% CI
**Parental demographics**								
Maternal age, years	≤19	18.2	**12.9**	**4.89–34.0**	**7.41**	**2.53–21.7**	**8.48**	**2.82–25.5**
	20–24	4.8	**2.95**	**1.66–5.25**	**2.19**	**1.18–4.06**	**2.38**	**1.27–4.46**
	25–29	3.1	**1.84**	**1.14–2.96**	1.39	0.84–2.30	1.48	0.89–2.46
	30–34	2.5	1.48	0.92–2.37	1.34	0.83–2.17	1.45	0.89–2.37
	35–39	1.7	Ref		Ref		Ref	
	≥40	2.8	1.68	0.75–3.75	1.71	0.75–3.88	1.74	0.76–3.99
Marital status	Married/living with partner	2.7	Ref		Ref		Ref	
	Single/divorced/widowed	8.0	**3.17**	**1.36–7.39**	2.45	0.94–6.40	2.32	0.88–6.07
Maternal employment status	Not working	2.8	Ref		Ref		Ref	
	Full-time	2.6	0.93	0.61–1.40	1.03	0.67–1.57	1.07	0.70–1.65
	Part-time	2.2	0.78	0.36–1.69	0.84	0.38–1.84	0.86	0.40–1.92
**Obstetrics history**								
Miscarriage	No	2.9	Ref		Ref		Ref	
	Yes	2.2	0.75	0.49–1.15	0.95	0.61–1.48	0.94	0.60–1.47
Induced abortion	No	2.8	Ref		Ref		Ref	
	Yes	2.8	0.98	0.54–1.77	0.83	0.45–1.54	0.84	0.45–1.56
Fertility treatment	No	2.8	Ref		Ref		Ref	
	Yes	2.3	0.81	0.49–1.34	0.80	0.47–1.38	0.76	0.44–1.31
**Infant characteristics**								
Age, weeks	≤12	2.6	1.00	0.50–1.97	1.00	0.50–2.00	1.07	0.53–2.18
	13–20	2.7	Ref		Ref		Ref	
	≥21	5.6	**2.20**	**1.06–4.57**	**2.13**	**1.00–4.54**	**2.20**	**1.02–4.74**
Sex	Boy	2.7	Ref		Ref		Ref	
	Girl	2.8	1.02	0.76–1.38	1.06	0.78–1.43	1.12	0.82–1.52
Birth weight, grams	<2500	3.9	1.50	0.95–2.39	1.26	0.72–2.18	1.20	0.68–2.10
	≥2500	2.6	Ref		Ref		Ref	
Gestational age, weeks	<37	4.3	1.63	0.97–2.76	1.36	0.72–2.55	1.27	0.67–2.40
	≥37	2.7	Ref		Ref		Ref	
Multiple births	Single	2.7	Ref		Ref		Ref	
	Twin	5.6	2.14	0.93–4.95	2.59*	0.97–6.92	2.68*	0.99–7.30
Delivery method	Vaginal	2.8	Ref		Ref		Ref	
	Cesarean	2.3	0.84	0.57–1.24	0.79	0.52–1.22	0.79	0.52–1.22
Birth order	First child	3.8	**2.28**	**1.66–3.14**	**2.11**	**1.49–2.98**	**1.94**	**1.36–2.77**
	Subsequent child	1.7	Ref		Ref		Ref	
**Household characteristics**								
Living with grandparents	Yes	2.0	0.71	0.43–1.17	0.63	0.35–1.12	0.61	0.34–1.09
	No	2.9	Ref		Ref		Ref	
House type	Detached house	2.1	1.03	0.61–1.75	**2.03**	**1.06–3.90**	**2.01**	**1.04–3.90**
	Apartment, 1st (ground) floor	2.0	Ref		Ref		Ref	
	Apartment, 2nd–9th floor	3.5	**1.72**	**1.05–2.84**	**2.04**	**1.22–3.40**	**2.00**	**1.20–3.35**
	Apartment, ≥10th floor	5.9	**3.01**	**1.29–7.03**	**5.50**	**2.25–13.5**	**5.90**	**2.38–14.6**
Number of rooms	1–2 rooms	4.0	1.30	0.85–1.97	1.22	0.79–1.88	1.18	0.76–1.84
	3 rooms	3.1	Ref		Ref		Ref	
	4 rooms	2.3	0.73	0.47–1.11	0.81	0.51–1.28	0.83	0.52–1.33
	≥5 rooms	1.8	**0.56**	**0.37–0.86**	0.75	0.42–1.32	0.78	0.43–1.39
Subjective economic status	Stable	2.4	Ref		Ref		Ref	
	Able to manage	2.7	1.15	0.82–1.61	1.11	0.79–1.57	0.94	0.66–1.34
	Difficult to manage or unstable	4.3	**1.87**	**1.22–2.87**	**1.82**	**1.16–2.87**	1.40	0.87–2.26
**Postpartum situation**								
Feeding status at 4 months	Breastfeeding only	2.5	Ref				Ref	
	Mixed	3.3	1.33	0.94–1.88			1.09	0.76–1.58
	Bottle-feeding only	2.7	1.07	0.65–1.77			0.72	0.42–1.22
Number of persons to consult with	0–5 persons	3.9	Ref				Ref	
	6–10 persons	2.1	**0.53**	**0.38–0.75**			**0.65**	**0.46–0.93**
	≥11 persons	2.1	**0.53**	**0.33–0.84**			0.74	0.45–1.21
Perception of frequency of infant crying	Not at all	1.4	Ref				Ref	
	Not so much	2.7	**1.98**	**1.28–3.06**			**1.87**	**1.19–2.92**
	A lot or to some extent	4.8	**3.66**	**2.35–5.69**			**2.96**	**1.87–4.69**
Postpartum depression by EPDS score	≥9	7.0	**3.17**	**2.22–4.51**			**2.37**	**1.60–3.52**
	≤8	2.3	Ref				Ref	

CI, confidence interval; OR, odds ratio; Ref, reference.

Bold signifies *P* < 0.05.

*P < 0.06.

^a^Model 1 adjusted for parental demographics (maternal age, marital status, and maternal employment status), obstetric history (miscarriage, induced abortion, and fertility treatment), infant characteristics (age in weeks, sex, birth weight, gestational age, multiple birth or not, delivery method, and birth order), and household characteristics (living with grandparents or not, house type, that is, detached house or apartment, and if apartment, level of living floor, number of rooms, and subjective socioeconomic status).

^b^Model 2 adjusted for covariates in Model 1 plus postpartum situation at 4 months (feeding status, number of persons to consult with, perception of frequent infant crying, and postpartum depression).

Table 5 shows the ORs of possible risk factors for either shaking or smothering, among all participants as well as stratified by Nagoya city and other cities in Aichi Prefecture. In Nagoya city, the prevalence of either shaking or smothering was 4.5%, which was significantly lower than the prevalence in other cities (6.0%) (*P* = 0.01). Among all participants, in addition to younger maternal age, mothers aged ≥40 years were 2.00 (95% CI, 1.11–3.60) times more likely to shake or smother their infant in comparison with mothers aged 35–39 years. Further, full-time working, later attendance at 4-month health checkup, primiparity, living in a detached house, living on the 2nd to 9th floor or 10th floor or higher in an apartment, living in a three-room house (in comparison to a four-room house), economic adversity, having a limited number of people to consult with, perceived larger amount of crying, and post-partum depression were significantly associated with infant abuse. These associations with shaking or smothering, especially the association of living on the 10th floor or higher in an apartment, were retained after stratification by Nagoya City and other cities.

**Table 5.** Odds ratio of parental demographics, obstetrics history, infant’s characteristics, household characteristics, and postpartum situation for either shaking or smothering at 4 month of age, adjusted model, stratified by Nagoya City and other cities

		Total (*n* = 6487)			Nagoya City (*n* = 2575)			Other cities (*n* = 3912)		
		
		Prevalence (%)	aOR^a^	95% CI	Prevalence (%)	aOR^a^	95% CI	Prevalence (%)	aOR^a^	95% CI
Total		5.4		4.9–6.0	4.5		3.7–5.3	6.0		5.3–6.8
**Parental demographics**										
Maternal age, years	≤19	21.1	**4.69**	**1.76–12.5**	33.3	**7.90**	**1.33–47.1**	16.7	**4.53**	**1.35–15.2**
	20–24	11.4	**3.11**	**1.99–4.86**	7.5	2.13	0.89–5.08	13.7	**3.62**	**2.11–6.21**
	25–29	6.2	**1.67**	**1.15–2.43**	6.4	1.82	0.96–3.45	6.1	**1.62**	**1.01–2.58**
	30–34	4.8	**1.51**	**1.06–2.17**	3.6	1.24	0.66–2.33	5.6	**1.74**	**1.11–2.70**
	35–39	3.2	Ref		2.7	Ref		3.5	Ref	
	≥40	6.0	**2.00**	**1.11–3.60**	4.5	1.90	0.65–5.53	7.2	**2.14**	**1.02–4.46**
Marital status	Married/living with partner	5.3	Ref		4.4	Ref		6.0	Ref	
	Single/divorced/widowed	13.3	1.86	0.88–3.95	14.3	2.43	0.75–7.88	12.5	1.76	0.62–4.97
Maternal employment status	Not working	5.3	Ref		4.8	Ref		5.7	Ref	
	Full-time	6.1	**1.41**	**1.05–1.90**	3.9	0.97	0.55–1.73	7.6	**1.72**	**1.21–2.43**
	Part-time	4.4	0.91	0.51–1.62	1.8	0.47	0.11–2.00	5.8	1.14	0.60–2.17
**Obstetrics history**										
Miscarriage	No	5.8	Ref		5.1	Ref		6.3	Ref	
	Yes	3.7	0.77	0.54–1.08	1.9	**0.42**	**0.20–0.91**	5.0	0.95	0.64–1.41
Induced abortion	No	5.4	Ref		4.7	Ref		5.9	Ref	
	Yes	5.8	0.96	0.62–1.48	2.8	0.48	0.18–1.25	7.9	1.16	0.70–1.92
Fertility treatment	No	5.6	Ref		4.8	Ref		6.1	Ref	
	Yes	4.4	0.83	0.56–1.24	2.5	0.53	0.23–1.23	5.5	0.96	0.60–1.51
**Infant characteristics**										
Age, weeks	≤12	7.3	1.51	0.96–2.36	5.9	1.66	0.74–3.69	8.3	1.40	0.80–2.47
	13–20	5.2	Ref		4.3	Ref		5.7	Ref	
	≥21	10.6	**2.27**	**1.26–4.07**	14.3	**4.00**	**1.02–15.7**	9.9	1.91*	0.997–3.67
Sex	Boy	5.6	Ref		4.1	Ref		6.6	Ref	
	Girl	5.3	1.03	0.82–1.28	4.9	**1.51**	**1.004–2.26**	5.5	0.87	0.66–1.14
Birth weight, grams	<2500	7.6	1.33	0.89–1.99	7.7	1.64	0.84–3.17	7.5	1.28	0.75–2.17
	≥2500	5.2	Ref		4.2	Ref		5.9	Ref	
Gestational age, weeks	<37	7.5	1.10	0.68–1.78	8.8	1.56	0.71–3.42	6.7	0.89	0.47–1.67
	≥37	5.3	Ref		4.3	Ref		5.9	Ref	
Multiple births	Single	5.4	Ref		4.4	Ref		6.1	Ref	
	Twin	7.5	1.44	0.61–3.38	10.9	3.00	0.86–10.5	4.9	0.76	0.20–2.83
Delivery method	Vaginal	5.7	Ref		4.7	Ref		6.3	Ref	
	Cesarean	4.3	0.74*	0.54–1.01	3.8	0.74	0.43–1.27	4.6	0.78	0.52–1.15
Birth order	First child	7.1	**1.51**	**1.18–1.94**	6.1	1.55	0.98–2.45	7.8	**1.54**	**1.13–2.09**
	Subsequent child	3.8	Ref		3.0	Ref		4.3	Ref	
**Household characteristics**										
Living with grandparents	Yes	4.9	0.74	0.50–1.10	3.7	0.74	0.32–1.69	5.4	0.74	0.47–1.16
	No	5.5	Ref		4.6	Ref		6.2	Ref	
House type	Detached house	4.6	**1.78**	**1.12–2.83**	2.9	1.79	0.70–4.62	5.2	1.67	0.96–2.91
	Apartment, 1st (ground) floor	4.8	Ref		3.2	Ref		5.7	Ref	
	Apartment, 2nd–9th floor	6.4	**1.58**	**1.12–2.25**	6.5	1.89	0.96–3.72	7.7	**1.54**	**1.00–2.35**
	Apartment, ≥10th floor	8.1	**3.65**	**1.74–7.64**	6.1	**3.40**	**1.12–10.4**	13.5	**5.64**	**1.84–17.2**
Number of rooms	1–2 rooms	7.5	1.09	0.79–1.50	7.0	1.07	0.62–1.84	7.8	1.08	0.71–1.64
	3 rooms	6.3	Ref		5.4	Ref		6.9	Ref	
	4 rooms	3.9	**0.66**	**0.46–0.95**	3.3	0.70	0.40–1.23	4.5	0.64	0.40–1.05
	≥5 rooms	4.1	0.78	0.51–1.18	2.7	0.76	0.34–1.72	4.7	0.80	0.48–1.33
Subjective economic status	Stable	4.4	Ref		3.7	Ref		4.8	Ref	
	Able to manage	5.8	1.11	0.86–1.43	4.8	1.03	0.65–1.62	6.5	1.15	0.84–1.57
	Difficult to manage or unstable	8.3	**1.49**	**1.05–2.12**	7.2	1.46	0.79–2.72	9.0	1.53	0.98–2.38
**Postpartum situation**										
Feeding status at 4 months	Breastfeeding only	4.7	Ref		4.3	Ref		4.9	Ref	
	Mixed	6.3	1.16	0.88–1.52	4.9	0.86	0.53–1.42	7.1	1.32	0.95–1.84
	Bottle-feeding only	6.4	1.02	0.71–1.47	4.6	0.69	0.34–1.39	7.4	1.27	0.82–1.95
Number of persons to consult with	0–5 persons	7.1	Ref		6.4	Ref		7.6	Ref	
	6–10 persons	4.7	**0.76**	**0.59–0.98**	3.4	**0.59**	**0.37–0.94**	5.5	0.84	0.61–1.14
	≥11 persons	3.7	**0.68**	**0.47–0.98**	3.3	0.74	0.40–1.39	3.9	0.68	0.43–1.09
Perception of frequency of infant crying	Not at all	2.3	Ref		1.2	Ref		3.1	Ref	
	Not so much	5.7	**2.48**	**1.77–3.47**	4.8	**4.32**	**2.13–8.76**	6.2	**2.12**	**1.43–3.13**
	A lot or to some extent	9.4	**3.79**	**2.68–5.37**	8.7	**7.11**	**3.45–14.7**	9.8	**3.13**	**2.08–4.72**
Postpartum depression EPDS score	≥9	12.8	**2.22**	**1.65–2.99**	10.9	**2.25**	**1.30–3.89**	13.9	**2.21**	**1.54–3.17**
	≤8	4.7	Ref		3.9	Ref		5.2	Ref	

CI, confidence interval; OR, odds ratio; Ref, reference.

*P < 0.06.

^a^aOR is odds ratio adjusted for adjusted for parental demographics (maternal age, marital status, and maternal employment status), obstetric history (miscarriage, induced abortion, and fertility treatment), infant characteristics (age in weeks, sex, birth weight, gestational age, multiple birth or not, delivery method, and birth order), and household characteristics (living with grandparents or not, house type, that is, detached house or apartment, and if apartment, level of living floor, number of rooms, and subjective socioeconomic status), and plus postpartum situation at 4 months (feeding status, number of persons to consult with, perception of frequent infant crying, and postpartum depression).

## DISCUSSION

Respective prevalence rates of shaking and smothering among mothers of 4-month-old infants in Aichi Prefecture, Japan were 3.9% and 2.7%, which are similar to the prevalence rates reported in western countries (eg, prevalence of shaking of 3.4% in the Netherlands^8^ and 2.6% in the United States,^15^ and prevalence of smothering of 1.6% in the Netherlands^8^). The frequencies of these abusive behaviors were significantly correlated, suggesting that they share the same or similar triggers, contexts, and risk factors. Indeed, a number of risk factors were similar, including young maternal age (<24 years old), living on the 10th floor or higher of an apartment complex, perceived excessive infant crying, and postpartum depression. Older infant age (more than 21 weeks compared to 13–20 weeks) was also associated with both shaking and smothering. This association could be due to the fact that mothers who visit the health checkup later than scheduled may be more prone to poor parenting, including abuse.

To our knowledge, the present study is the first to report on the prevalence of shaking and smothering among a large, prefecture-wide population sample in Japan, where small living arrangements could increase frustration due to infant crying (a known trigger for shaking and a possible trigger for smothering). Apartment complexes are common^9^ in Japanese housing, and detached houses are small compared to houses in western countries. On the other hand, infant crying is relatively accepted in Japan. There is a Japanese proverb, “a crying baby grows well”, which leads mothers to positively embrace infant crying. These factors may balance each other and explain why the prevalence of shaking in our study was similar to that reported in previous studies in the Netherlands (1.3% and 3.4% of parents of 3- and 6-month-old infants^8^) and the United States (2.6% of parents of <2-year-old children^15^).

Further, consistent with previous studies in western countries,^3^^,^^4^ perceived infant crying as more than ‘not at all’ was associated with shaking. In the Dutch study, parents who were worried about their child crying sometimes or frequently were 3.05 times more likely to shake their infant than those who never worried about their child crying.^8^ Although crying frequency was not assessed objectively in our study, it is important to note that the caregiver’s perception of crying frequency is relevant for shaking.

Further, our findings reveal that living on or above the 2nd floor of an apartment complex, especially on the 10th floor or higher, is an independent risk factor for shaking and smothering. The higher risk for those living on the 10th floor or higher may be because the higher floors are quieter, with less traffic noise.^16^^,^^17^ Mothers might be more sensitive to the sound of infant crying, which may induce shaking or smothering. Moreover, mothers living on a higher floor may be reluctant to go out,^18^ which might contribute to increased stress from crying or parenting in general. Further studies that investigate noise level by floor, activities with infants (eg, going out for a walk and stress of parenting), and frustration due to crying are warranted. In addition, living in a detached house was a risk factor for shaking and smothering after adjustment for subjective socioeconomic status. This association might be due to worrying about bothering neighbors due to infant crying, because people living in detached houses are more likely to have enriched neighborhood relationships.^19^

We also found that younger mothers (<25 years old) were at higher risk for shaking and smothering than older ones. This finding is consistent with that of previous studies assessing the risk factors for hospitalized AHT/SBS cases^5^ or infant homicide cases in the United States.^20^ Based on this evidence, we recommend defining a young mother at high risk for shaking or smothering their infant as a woman aged 24 years or less (ie, not only less than 20 years of age).

In addition, we found that mothers who are 40 years or older are twice as likely to shake or smother their infant as those aged 35–39 years. This could be due to older mothers being more easily physically stressed, especially when they are dealing with their first child. Further, it is likely that older mothers, who are more likely to have been working full-time and are used to controlling their job, find child-rearing more stressful than younger ones.

We also found that subjective economic status was associated with shaking, which is consistent with previous studies on infant shaking.^21^^,^^22^ Past research that reported an association between poverty and SBS/AHT was based on neighborhood deprivation, not on individual socioeconomic status.^21^^,^^22^ Thus, our finding that perception of poverty measured at the individual level can be a marker to detect risk of shaking is novel and suggests that promotional materials on shaking or smothering prevention that target poor families are needed.

The association between postpartum depression and shaking is consistent with previous studies investigating the association between postpartum depression and stress due to crying.^23^^,^^24^ Crying, especially excessive or inconsolable crying, is a trigger for shaking^3^^,^^4^ and smothering by caretakers,^8^ who behave this way in an attempt to stop the infant from crying.^7^ Further, we confirmed that postpartum depression *per se* is an independent risk factor for shaking and smothering, regardless of the perceived amount of crying. Depressed mothers might have lower thresholds of patience for infant crying.

We also found that maternal full-time working status was an independent risk factor for shaking, but not for smothering. The increased risk of shaking is likely due to accumulation of stress during early infancy: working full time in addition to taking care of a 4-month-old infant induces a large amount of stress^25^ and increases the risk of shaking. We failed to detect an association between full-time working and smothering, which has also not been reported in previous studies; however, this could be due to mothers who work full-time having higher education attainment and being more likely to know the danger of smothering, as it is easy to imaging that smothering an infant’s mouth could stop his/her breathing.

The inverse association between experience of miscarriage and shaking could be due to mothers who have experienced miscarriage parenting differently from mothers without such experience, although research on this association is scare.^26^ It has been reported that women who have experienced infant loss tend to be distant with children born later,^27^ which may be associated with the protective effect of experience of miscarriage against shaking.

Whatever the mechanism, future prevention efforts should target mothers who have risk factors for shaking and smothering that are amenable to change. For example, public health nurses should approach mothers who are at risk of postpartum depression (eg, those who had a positive screen using the EPDS) and provide emotional support. Further, mothers who are living on higher floors should be carefully monitored for shaking and smothering, and they should be encouraged to find opportunities to leave their house more often, especially if frustrated by their infant’s crying. Educational material on how to deal with infant crying to prevent shaking and smothering should be provided to these high-risk mothers, which could be delivered through public health home visit programs, such as Home Visit Service for Newborns or Home Visit Project for All Infants.^28^ The effectiveness of these activities should also be evaluated in terms of prevention of infant abuse.

Several limitations to the present study need to be mentioned. First, shaking and smothering were self-reported, not based on more objective measurements, such as video recordings or diary records. However, this is similar to previous studies that have used self-administered questionnaires to assess the prevalence of shaking and smothering.^8^^,^^29^ In addition, we confirmed the robust association between self-reported shaking and smothering and amount of perceived crying, which is one of the established risk factors,^2^^–^^4^ suggesting that self-reported shaking and smothering measurement had good criterion-related validity. Second, cases of shaking and smothering might have been misclassified, although we attempted to reduce this error by clearly defining ‘shaking’ and ‘smothering’ in the questionnaire. The interpretation of shaking might be different in Japanese culture^29^; for this reason, we defined ‘shaking’ as “violent shaking while the infant is crying”. Third, we assessed the prevalence of shaking and smothering in one prefecture, which is not a representative sample of Japan and may influence the generalizability of our findings. Therefore, further study is warranted to replicate the prevalence of and risk factors for shaking and smothering, using larger representative sample populations in Japan. Fourth, although we conducted a population-based survey, some participants did not respond to the survey. This might have resulted in under- or over-estimation of the prevalences of shaking and smothering if these behaviors were more or less prevalent among non-respondents. In this study, we excluded participants who did not provide valid responses on shaking or smothering; since these excluded participants were of younger maternal age and more likely to be primiparous than the included participants, it is likely that we underestimated our findings due to selection bias. Thus, further study is warranted to investigate the shaking and smothering behaviors among caregivers of 4-month-old infants at the time of routine postnatal health checkups.

In conclusion, the prevalence of self-reported shaking and smothering in Japan was consistent with that in western countries. Risk factors for both shaking and smothering were younger maternal age, living on the 10th floor of an apartment complex or higher, perception of excessive infant crying, and postpartum depression. Hence, we suggest that educational materials on how to manage stress and frustration due to infant crying and the dangers of shaking and smothering be provided to high-risk women during prenatal or postnatal care. Further study is needed to replicate and elucidate the risk factors for shaking and smothering in other cultures.

## ONLINE ONLY MATERIAL

Abstract in Japanese.
