# Supplementary material for: Self-Reported Prevalence and Risk Factors for Shaking and Smothering Among Mothers of 4-Month-Old Infants in Japan
Source: J Epidemiol. 2016 Jan 5;26(1):4–13. doi: 10.2188/jea.JE20140216 (PMC4690735; doi:10.2188/jea.JE20140216)
Supplement: Abstract in Japanese. [file je-26-004-s001.pdf]

日本における 4 か月の乳児をもつ母親における揺さぶりおよび口塞ぎの自己申告存在割合およびリスク因子

藤原武男[1、2]、山岡祐衣[3]、森崎菜穂[1]

[1]国立成育医療研究センター研究所社会医学研究部

[2]三重大学大学院医学系研究科成育社会医学分野

[3]筑波大学医学医療系ヘルスサービスリサーチ分野

背景 : 日本の人口ベースのサンプルを用いて、4 か月の乳児をもつ母親の揺さぶりと口塞ぎの存在割合を推定し、リスク因子を明らかにすること。

方法 : 愛知県 45 市の 4 か月健診を受診した母親に質問紙調査を実施した(N=6,487, 有効回答率: 66.8%)。その質問紙で、過去 1 か月間の揺さぶりおよび口塞ぎの頻度、母親・子の特性、世帯の状況について調査した。そして、これらリスク因子と揺さぶり、口塞ぎ、揺さぶりまたは口塞ぎとの関連をロジスティック回帰分析によって検討した。

結果 : 自己申告による過去 1 か月の 1 回以上の揺さぶり、口塞ぎ、そのどちらかの存在割合は、それぞれ 3.9% (95%信頼区間 : 3.5%-4.4%)、2.7% (95%信頼区間 : 2.3%-3.1%), 5.4% (95% 信頼区間 : , 4.9%-6.0%)であった。揺さぶりまたは口塞ぎのリスク因子は、母親の年齢が 34 歳以下 (特に 24 歳以下) または 40 歳以上、母親が常勤で勤務していること、4 か月健診の受診月齢が遅いこと、初産、一戸建て居住、2階以上の集合住宅 (特に１０階以上の集合住宅) の居住、主観的経済状況の悪さ、泣きの量を多いと感じること、産後うつ、であった。保護因子としては部屋の数が４つあること、そして相談できる人の数が多いこと、であった。

結論 : 日本における自己申告の揺さぶりおよび口塞ぎの存在割合は欧米諸国と同等であった。この研究結果が、揺さぶりや口塞ぎといった乳児虐待のリスクを持つ母親を特定することに役立つかもしれない。
